# Investigation into the efficacy of fully automated three-dimensional echocardiographic quantification software (3D Auto RV) for assessing right ventricular systolic function in patients suffering from chronic mountain sickness

**DOI:** 10.3389/fcvm.2026.1831242

**Published:** 2026-09-10

**Authors:** Jiahui Zhao, Xuebo Zhao, Xianxia Chen

**Affiliations:** 1 Department of Ultrasound Medicine, Qinghai Provincial People’s Hospital, Xining, China; 2 Department of Ultrasound Medicine, Dazhou Central Hospital, Dazhou, China

**Keywords:** chronic mountain sickness, echocardiography, pulmonary hypertension, right ventricular fractional area change, right ventricular quantification, right ventricular systolic function, three-dimensional echocardiography

## Abstract

**Objective:**

To evaluate the efficacy of fully automated three-dimensional echocardiographic right ventricular quantification software (3D Auto RV) for assessing right ventricular (RV) systolic function in patients with chronic mountain sickness (CMS).

**Methods:**

In this prospective study (December 2023–March 2025), 32 CMS patients and 32 propensity score-matched controls (by age and sex) underwent 3D echocardiography. Conventional echocardiography measured main pulmonary artery diameter (MPA) and estimated pulmonary artery systolic pressure (PASP). The 3D Auto RV software was employed to obtain tricuspid annular plane systolic excursion (TAPSE), RV ejection fraction (RVEF), RV fractional area change (RVFAC), septal longitudinal strain (SLS), free-wall longitudinal strain (FWLS), and tissue Doppler S’. Intraclass correlation coefficients (ICC) were calculated for intra- and inter-observer agreement.

**Results:**

The CMS group exhibited significantly lower RVEF, TAPSE, RVFAC, SLS, FWLS, and S’, alongside higher PASP and MPA, compared with controls (all *P* < 0.001). Spearman correlation showed RVEF was positively correlated with SLS (r = 0.890, *P* < 0.01) and FWLS (r = 0.840, *P* < 0.01), and negatively correlated with PASP (r = −0.707, *P* < 0.01). ROC analysis revealed excellent diagnostic efficacy: RVEF (AUC 0.982, cut-off 46.7%), RVFAC (AUC 0.970, cut-off 36.75%), TAPSE (AUC 0.965, cut-off 16.2 mm), SLS (AUC 0.975, cut-off 21.8%), and FWLS (AUC 0.968, cut-off 19.85%). All parameters demonstrated good to excellent repeatability, with intra- and inter-observer ICCs exceeding 0.85.

**Conclusion:**

3D Auto RV provides an accurate, reproducible, and feasible method for assessing RV systolic function in CMS patients, establishing a foundation for its routine clinical use.

## 1 Introduction

Researchers have determined that acute exposure to high-altitude environments (altitude >2,500 m) can lead to acute mountain sickness, high-altitude cerebral edema, and high-altitude pulmonary edema. Prolonged exposure (>3 months) often results in chronic mountain sickness (CMS) (1), characterized by erythrocytosis. Key symptoms include sleep disorders, dizziness, headaches, tinnitus, sensory abnormalities, reduced exercise tolerance, physical weakness, and mental fatigue. As CMS progresses, it may cause pulmonary hypertension and heart failure. The primary impact of high altitude stems from low atmospheric pressure, reducing the partial pressure of oxygen in inhaled air, thereby decreasing oxygen bioavailability in organs, tissues, and cells. In response to high-altitude conditions, the body triggers compensatory mechanisms, such as pulmonary arteriolar endothelial cell proliferation, leading to arteriole contraction, spasm, and pulmonary artery remodeling (2–5), which increases pulmonary artery pressure. This pressure is transmitted to the right heart, raising the right ventricle's afterload and creating a vicious cycle. Initially, the right heart compensates by thickening and increasing its workload to maintain normal function. High-altitude heart disease (HAHD) (6, 7) is marked by pulmonary hypertension (PH) and right ventricular hypertrophy (RVH) (8, 9). Erythrocytosis, a compensatory response to long-term high-altitude exposure, is influenced by genetic and susceptibility factors. Chronic hypoxemia activates the HIF pathway, reducing pulmonary ventilation and exacerbating hypoxia. Concurrently, long-term oxidative inflammatory nitrous acid stress (OXINOS) triggers inflammation and decreases nitric oxide (NO) bioavailability, causing vascular endothelial dysfunction, microvascular damage, and neurovascular unit dysfunction. In the cardiovascular system, this manifests as increased hemoglobin concentration and thrombus formation, potentially leading to pulmonary hypertension. If a patient exhibits HAHD symptoms like pulmonary hypertension and right ventricular hypertrophy, along with erythrocytosis, CMS can be diagnosed (10–13). As the disease advances, right-heart failure occurs when the right ventricle can no longer handle the afterload pressure. Studies indicate that right-heart failure independently influences adverse cardiovascular events (14, 15). Epidemiological data suggest that approximately 40 million people worldwide experience short-term high-altitude exposure, while over 80 million reside above 2,500 meters long-term (16, 17). Consequently, CMS is a significant public health issue in high-altitude areas, affecting many residents and requiring urgent attention. To enhance the health of high-altitude populations, it is crucial to swiftly and accurately assess CMS patients' right-heart function. Currently, two primary methods exist for evaluating right-heart function: non-imaging and imaging techniques. The pulmonary artery catheterization floating experiment is the gold standard among non-imaging methods but is limited by its invasiveness. Echocardiography, an imaging method, is favored in clinical practice for its simplicity and accuracy. Traditional two-dimensional echocardiography is commonly used to assess right-heart structure and function (18), but it has limitations due to the right ventricle's unique structure. Anatomically, the right ventricle is located behind the sternum, and the ultrasound beam is obstructed by bone, requiring the operator to adjust the probe angle and patient position for optimal detection. Additionally, the right ventricle's crescent-like, irregular structure (19, 20) differs from the conical left ventricle, necessitating continuous section changes and comprehensive evaluation, which depend heavily on the operator's skill, limiting echocardiography's widespread clinical use for right-ventricular function assessment. However, advancements in echocardiography and its integration with artificial intelligence have led to the development of Real-time three-dimensional ultrasound right-ventricular quantitative software (3D Auto RV). This technology shows a high correlation with cardiac magnetic resonance, the gold standard for right-heart function measurement, indicating its accuracy in evaluating right-heart function parameters (21–25). This study aims to use 3D Auto RV to assess the right-ventricular systolic function in high-altitude heart disease patients, explore its application value in CMS patients, aid in establishing precise diagnostic criteria, optimize clinical treatment pathways, and provide new evidence-based medical insights for individualized CMS diagnosis and treatment.

## 2 Materials and methods

### 2.1 Sample size estimation

**Figure 1:**
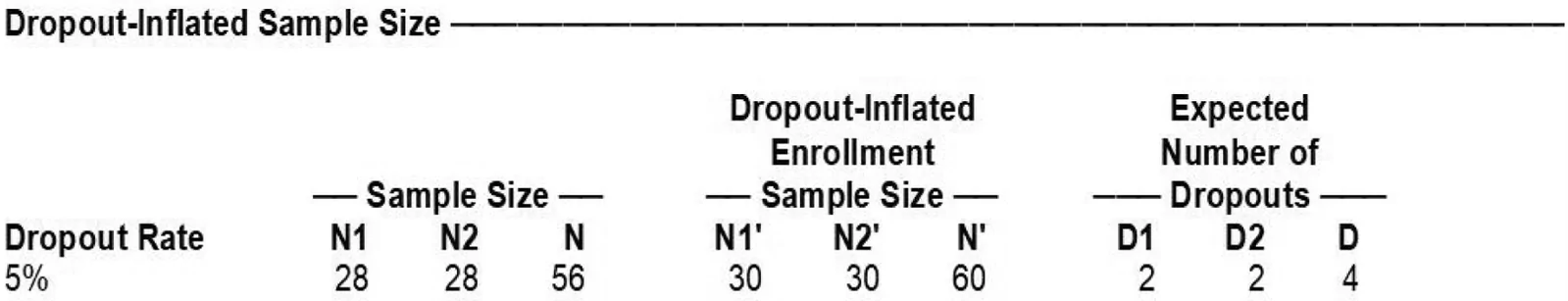
Sample size calculation using PASS software.

The sample size was estimated using PASS 2025 (NCSS, LLC, Kaysville, UT, USA), employing the confidence interval method for the area under the receiver operating characteristic curve (AUC). A two-sided 95% confidence interval was specified. Assuming a 1:1 allocation ratio between the observation and control groups, and based on findings from reference (25) and our pilot study, we set the expected AUC at 0.95 and the anticipated confidence interval width at 0.12. Accounting for a 5% dropout rate, the calculation yielded a required minimum of 30 patients per group (Figure 1).

### 2.2 Research subjects

This study is a single-center, case-control prospective investigation. We selected 32 patients with chronic mountain sickness(CMS) who received either outpatient or inpatient treatment at Qinghai Provincial People's Hospital from December 2023 to March 2025 as the observation group (CMS group). To ensure comparability and minimize selection and confounding biases during the control group matching process, we matched patients based on age and gender. We employed the propensity score matching method, using the Mahalanobis distance for 1:1 matching. Propensity score matching (PSM) was used to control for confounding bias. A logistic regression model was constructed with age and sex as covariates to estimate the propensity score for each participant. Patients were matched in a 1:1 ratio using nearest-neighbor matching, with a caliper width set at 0.2 times the standard deviation of the propensity scores. Of the initial 56 CMS patients and 56 healthy controls, 32 pairs were successfully matched, while 24 CMS patients and 24 controls were excluded because they could not be successfully matched. After matching, there were no significant differences between the two groups in age (CMS: 50.1 ± 9.3 years vs. control: 47.0 ± 9.5 years, *P* = 0.90) or sex (CMS: 19 males vs. control: 18 males, *P* = 0.62). The standardized mean differences (SMDs) after matching were <0.1 for both age and sex, indicating good balance in baseline characteristics. All CMS patients were from regions in Qinghai Province at altitudes of 2,500–4,500 m, with a mean residential altitude of 3,200 m and a duration of residence >15 years. All CMS patients underwent assessment using the Qinghai CMS scoring scale, with a median score of 12 (IQR, 2.75); among them, 2 had mild CMS (score 6–10), 18 had moderate CMS (score 11–14), and 12 had severe CMS (score >15). At the time of echocardiographic examination, none of the patients had received medications that could affect right ventricular function, such as acetazolamide or vasodilators, for at least 2 weeks (washout period ≥2 weeks). This study received approval from the Medical Ethics Committee of our hospital [Approval Number: Qinghai Provincial People's Hospital Research Ethics Review (2023)—148]. The procedures adhered to the Helsinki Declaration of 2013. All patients were informed about the study and provided signed informed consent.

Inclusion Criteria: Based on the Qinghai criteria for chronic mountain sickness, CMS is diagnosed in the presence of excessive erythrocytosis together with a total score ≥6 points. The specific details are outlined below (Table 1).

**Table 1:** The Qinghai CMS score system.

Items	Degree	Scores
Dyspnea or palpitations	None	0
	Mild	1
	Moderate	2
	Severe	3
Sleep	Normal	0
	It is not as good as normal	1
	Prolonged wakefulness and poor sleep	2
	Difficulty falling asleep	3
Cyanosis	None	0
	Mild	1
	Moderate	2
	Severe	3
Local paresthesia	None	0
	Mild	1
	Moderate	2
	Severe	3
Headache	None	0
	Mild	1
	Moderate	2
	Severe	3
Tinnitus	None	0
	Mild	1
	Moderate	2
	Severe	3
Hemoglobin (Male)	18–21 g/dL	0
	≥21 g/dL	3
Hemoglobin (Female)	16–19 g/dL	0
	≥19 g/dL	3

CMS diagnosis requires excessive erythrocytosis and total scored ≥6 points.

Exclusion criteria include: (1) Patients diagnosed with other organic heart conditions, such as congenital heart disease or valvular heart disease. (2) Chronic pulmonary heart disease; (3) Patients with right heart failure caused by other reasons; (4) Patients with poor ultrasound images;.

### 2.3 Instruments and methods

#### 2.3.1 Echocardiographic examination

Patients underwent examination with a Philips EPIQ 7C color ultrasound diagnostic system, utilizing S5-1 and X5-1 probes (frequency 1−5 MHz). Each subject was positioned in the left lateral decubitus position and connected to an electrocardiogram for synchronization. The probe was aimed at the right ventricle to capture 2DE images from the Apical four-chamber view (4AC). It is crucial to display the maximum right ventricular internal diameter to prevent right ventricular shrinkage. Routine 2DE ultrasound was employed to measure the main pulmonary artery diameter (MPA) and estimate the pulmonary artery systolic pressure (PASP) using tricuspid regurgitation velocity.

#### 2.3.2 Three-dimensional imaging

Activate the Philips EPIQ 7C color ultrasonic diagnostic system with the X5-1 matrix probe to capture echocardiograms from the patient's apical conduction area. Before starting, adjust the gain, compression, and time-gain compensation settings to minimize signal attenuation and enhance endocardial visualization. While the patient holds their breath, use the X5-1 probe to acquire a focused dataset of the 4-chamber right ventricular heart view (RVFV), incorporating wide-angle, single-slice, and high frame rate options (Heart Model mode on the EPIQ system). In full-volume mode, centered on the RV at the 4AC view, obtain 3DE images at a frame rate (FR) of 23 (IQR [20–32]) frames per second. Collect four to five consecutive cardiac cycles during a single breath-hold. Ensure the entire right ventricular cavity is included in the scanning volume throughout the cardiac cycle. Adjust the imaging depth and sector width to maximize the frame rate.

#### 2.3.3 3D Auto RV analysis and parameter measurement

**Figure 2:**
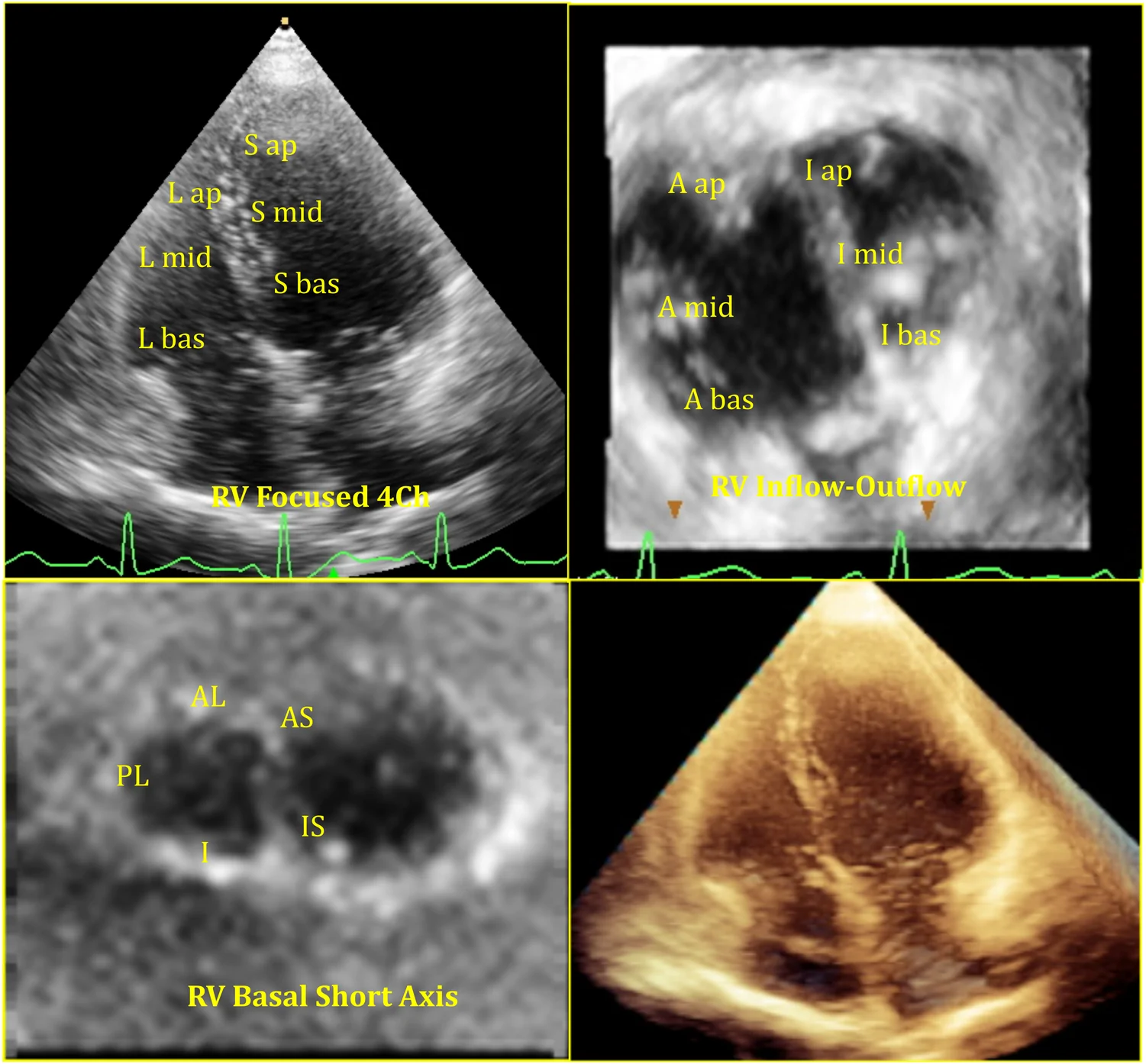
Under full-volume mode, 4Ch focused view (top left), the RV basal short axis view (bottom left), and inflow–outflow tract view (top right) were acquired.

**Figure 3:**
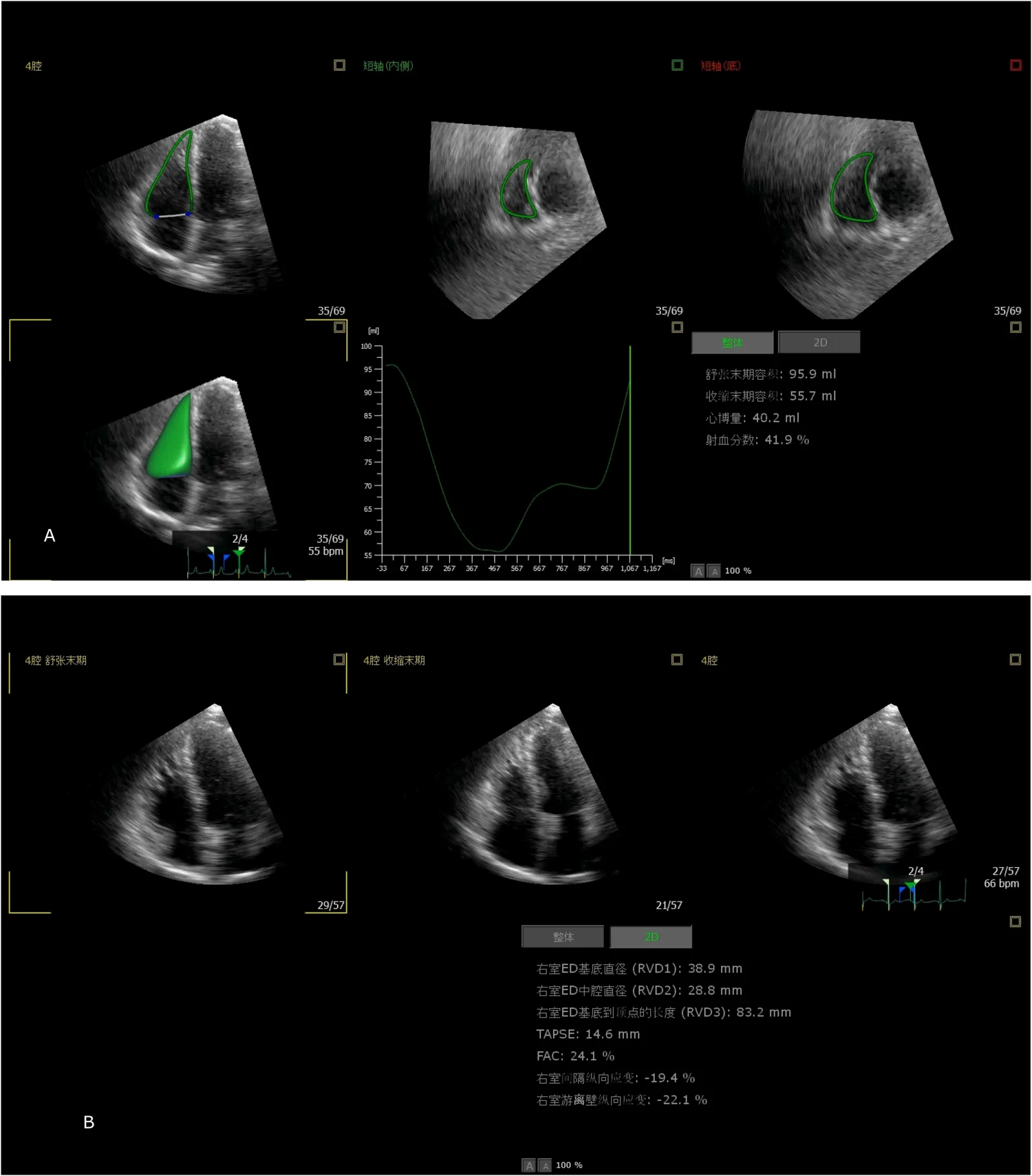
(**A**) Global right ventricular functional parameters obtained by 3D Auto RV analysis, including RVEF. (**B**) Two-dimensional right ventricular functional parameters obtained by 3D Auto RV analysis, TAPSE, FAC, SLS, FWLS.

Experienced echocardiography experts analyzed three-dimensional images to ensure dataset quality. From the RV-centered 4AC view in full-volume mode, images of the right ventricular basal short axis, 4AC, and inflow-outflow tract were extracted. The right ventricle was divided into 17 segments: basal (segments 1−6), mid (segments 7–12), apical (segments 12–16), and the non-cavitary apical segment (segment 17). These included the anterior basal wall (A bas), lateral basal wall (L bas), inferior basal wall (I bas), anteroseptal wall (AS), anterolateral wall (AL), septal basal wall (S bas), lateral mid wall (L mid), anterior mid wall (A mid), inferior mid wall (I mid), septal mid wall (S mid), anteroseptal mid wall, anterior apical wall (Aap), inferior apical wall (Iap), lateral apical wall (Lap), septal apical wall (Sap), and apex (apx). Each segment's visual quality was scored: 0 for invisible, 1 for partially visible, and 2 for visible, with scores ranging from 0 to 34. A score above 23 indicated good quality, 18–23 fair quality, and below 18 poor quality (Figure 2) (22). The 3D Auto RV analysis software was used for RV quantitative analysis, as shown in Figures 3. The Hough transform facilitated automatic segmentation by tracking and locating the four cardiac chambers and identifying the right ventricle's endocardial border for three-dimensional speckle tracking analysis. This automatic segmentation relied on a deformable model, accurately aligning cardiac chamber boundaries with structures and optimizing edge recognition by separating chambers from surrounding tissues. If identification and tracking were unsatisfactory, experienced doctors manually adjusted parameters to minimize discrepancies between the detected shape and the chamber's true contour. Three-dimensional quantitative parameters of the right ventricle were obtained, including TAPSE, right ventricular ejection fraction (RVEF), RVFAC, longitudinal strain of the ventricular septum (SLS), longitudinal strain of the free wall (FWLS), and S’.

*Right ventricular end—diastolic volume (RVEDV): 95.9 mL; Right ventricular end—systolic volume (RVESV): 55.7 mL; Right ventricular stroke volume (RVSV): 40.2 mL; Right ventricular ejection fraction (RVEF): 41.9%; Right ventricular basal segment diameter (RVD1): 38.9 mm; Right ventricular mid—diameter (RVD2): 28.8 mm; Right ventricular length from base to apex (RVD3): 83.2 mm; Tricuspid annular plane systolic excursion (TAPSE): 14.6 mm; Right ventricular fractional area change (FAC): 24.1%; Septal longitudinal strain (SLS):—19.4%; Right ventricular free - wall longitudinal strain (FWLS):—22.1%.

#### 2.3.4 Repeatability test

To assess the repeatability of the three-dimensional automatic software in measuring right ventricular (RV) systolic function, both inter-observer and intra-observer agreements were evaluated. For intra-observer agreement, the same doctor reanalyzed all images after a 2-week interval. For inter-observer agreement, a different doctor, blinded to the initial results, analyzed the images after 2 weeks. The interclass correlation coefficient (ICC) was used for analysis, with an ICC greater than 0.75 indicating good agreement.

### 2.4 Statistical methods

Data analysis was conducted using SPSS 26.0. The Shapiro–Wilk test assessed the normality of continuous variables. Variables that met normality were expressed as mean ± standard deviation (SD); those that did not were expressed as median and interquartile range (IQR). Categorical variables were reported as frequencies (percentages), and the chi-square test was applied to count data. For group comparisons of variables meeting normality and homogeneity of variance, the independent-samples t-test was employed. For non-normally distributed data, the Friedman analysis and Wilcoxon test were used for group comparisons. Correlations between variables were evaluated using Spearman's or Pearson's correlation analysis. Diagnostic efficacy was described using the receiver operating characteristic curve (ROC), and the intraclass correlation coefficient (ICC) assessed robustness and reproducibility. To control for potential confounding factors and to examine the independent effects of each variable, multiple linear regression models were constructed using the Enter (forced entry) method, with echocardiographic parameters (including RVEF) as dependent variables and potential confounders (BMI, smoking history, and diabetes history) as independent variables. Results were reported as unstandardized regression coefficients (B), standardized coefficients (Beta), 95% confidence intervals (CIs), and *P*-values. For the multiple between-group comparisons involving the eight echocardiographic parameters presented in Table 2, the Holm–Bonferroni sequential correction procedure was applied to avoid inflation of Type I errors (false positives). After correction, a *P*-value <0.05 was considered statistically significant.

**Table 2:** Comparative analysis of 3D Auto RV ultrasound parameters between the two groups.

Parameter	Control group	CMS group	*Z*	*P*
RVEF (%)	57.500 [54.125, 62.850]	37.650 [36.425, 42.675]	−6.627	<0.001
RVFAC (%)	39.000 [37.825, 39.850]	28.650 [23.975, 35.275]	−6.467	<0.001
TAPSE (mm)	18.600 [17.325, 19.575]	13.100 [11.375, 14.575]	−6.394	<0.001
SLS (%)	25.700 [23.875, 26.375]	13.050 [9.600, 19.825]	−6.528	<0.001
FWLS (%)	26.250 [24.150, 28.475]	16.900[15.750, 18.300]	−6.441	<0.001
MPA (mm)	21.000 [19.000, 23.750]	26.000 [24.250, 27.000]	−5.421	<0.001
S’	14.200 [13.100, 15.650]	10.150 [9.450, 10.600]	−6.462	<0.001
PASP (mmHg)	19.500 [17.000, 25.500]	40.000 [31.250, 47.500]	−6.719	<0.001

All *P*-values in the table were adjusted using the Holm–Bonferroni method (with adjusted significance thresholds ranging from 0.00625 to 0.05). After correction, all *P*-values for the between-group comparisons remained <0.05.

## 3 Results

### 3.1 Analysis of general data

#### 3.1.1 General characteristics of the two groups of people

As shown in Table 3, a comparison of the general data characteristics between the two patient groups revealed significant differences in systolic blood pressure (SBP) and heart rate. However, no significant differences were observed in other general data, including gender, age, height, weight, BMI, BSA, and frame rate. This suggests that the two groups were well-balanced in terms of baseline characteristics. Additionally, the distribution of image quality did not differ significantly between the groups (*χ*^2^ = 0.6308, *p* = 0.7295).

**Table 3:** General characteristics of the two groups of people..

Characteristic	Control group (*n* = 32)	CMS group (*n* = 32)	*p*
Male sex	18	19	0.800
Age, years	47.0 ± 9.5	50.1 ± 9.3	0.198
Height,cm	169[161,171]	173[161,176]	0.115
Body weight,kg	61.2 ± 8.0	64.8 ± 9.2	0.104
BMI,kg/m^2^	22.0 ± 2.3	22.6 ± 2.1	0.240
BSA,m^2^	1.7 ± 0.1	1.8 ± 0.2	0.105
SBP, mmHg	121.0 ± 9.7	139.0 ± 6.6	<0.001
DBP, mmHg	74.4 ± 5.8	78.6 ± 5.0	0.003
HR, bpm	73.5 ± 6.2	81.2 ± 6.2	<0.001
Frame rate, vps	25.6 ± 3.3	24.5 ± 2.1	0.121
Image quality	Control group	CMS group	
Excellent(%)	13 (41)	11 (34)	0.870
Acceptable(%)	17 (53)	19 (59)	
Poor(%)	2 (6)	2 (7)	

Abbreviations: BMI, body mass index; BSA, body surface area; HR, heart rate; SBP/DBP, systolic blood pressure/diastolic blood pressure.

#### 3.1.2 Comparison of laboratory test results between the two patient groups

Table 4 illustrates significant differences in RBC (red blood cell count), HGB (hemoglobin level), and HCT (hematocrit) between the two groups. CMS patients exhibited notably higher RBC, HGB, and HCT levels compared to the control group.

**Table 4:** Laboratory data comparison between Two cohorts.

Characteristic	Control group (*n* = 32)	CMS group (*n* = 32)	*p*
RBC(×10¹²/L)	5.2 ± 0.4	7.1 ± 0.4	<0.001
HGB(g/L)	144 [138, 152]	213 [197, 231]	<0.001
HCT(%)	48.9 [43.6, 52.9]	64.7 [71.4, 74.0]	<0.001

### 3.2 Comparative analysis of 3D Auto RV ultrasound parameters between the two groups

The results indicated that the RVEF (%) in the CMS group was significantly lower than in the control group, measuring 37.650 [36.425, 42.675] compared to 57.500 [54.125, 62.850]. Additionally, the PASP in the CMS group was significantly higher, at 40.000 [31.250, 47.500], compared to 19.500 [17.000, 25.500] in the control group. For the other parameters (RVFAC, TAPSE, SLS, FWLS, MPA, S'), the CMS group consistently showed significantly lower values than the control group.

### 3.3 Multiple linear regression analysis adjusting for confounding factors

To assess the independent associations of BMI, smoking history, and diabetes history with RVEF, we performed multiple linear regression (Enter method) with RVEF as the dependent variable. The overall model was not significant (F = 0.226, *P* = 0.878; adjusted R^2^ = –0.038), indicating negligible explanatory power. After adjustment, none of the three variables were independently associated with RVEF: BMI (*β* = 0.118, *P* = 0.414, 95% CI: −0.831 to 1.992), smoking history (*β* = –0.006, *P* = 0.962, 95% CI: −6.530 to 6.228), or diabetes history (*β* = –0.049, *P* = 0.737, 95% CI: −9.506 to 6.758) (Table 5).

**Table 5:** Multivariable linear regression analysis of factors associated with RVEF.

Variables	B	SE	Standardized β	t	*P*-value	95% CI for B
(Intercept)	37.255	14.512	–	2.567	0.013	8.227–66.282
BMI (kg/m^2^)	0.580	0.706	0.118	0.822	0.414	−0.831–1.992
Smoking history (yes vs. no)	−0.151	3.189	−0.006	−0.047	0.962	−6.530–6.228
Diabetes history (yes vs. no)	−1.374	4.065	−0.049	−0.338	0.737	−9.506–6.758

B, unstandardized regression coefficient; SE, standard error; β, standardized regression coefficient; CI, confidence interval; BMI, body mass index; RVEF, right ventricular ejection fraction.

Model fit: F(3, 60) = 0.226, *P* = 0.878; Adjusted R^2^ = −0.038. All variables were entered using the Enter method.

### 3.4 Correlation analysis of 3D auto RV ultrasound parameters

**Figure 4:**
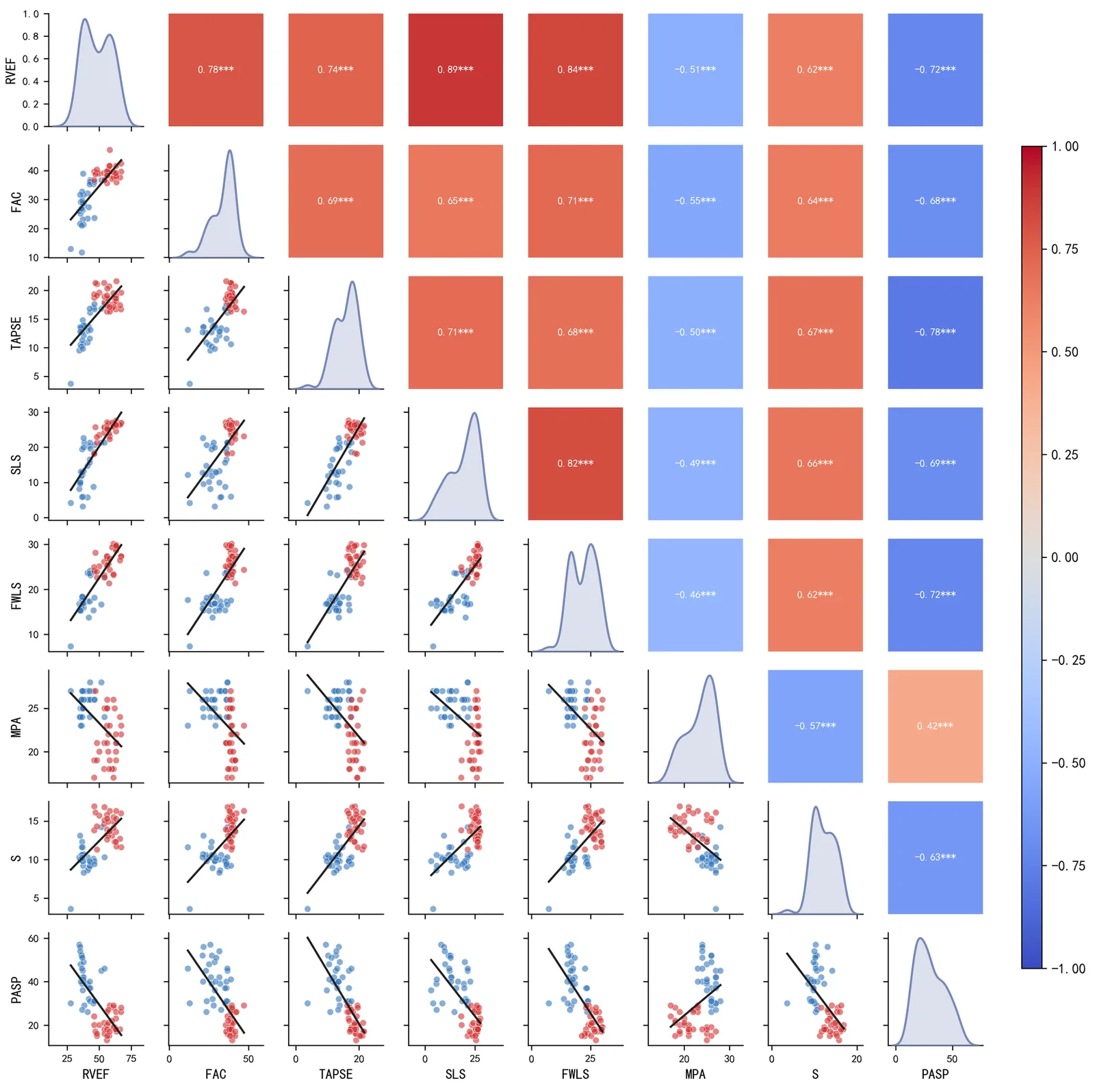
Correlation matrix of echocardiographic parameters in CMS and control patients.

As illustrated in Figure 4, the results indicated a significant positive correlation between RVEF and the right ventricular functional parameters, SLS (r = 0.890, *P* < 0.01) and FWLS (r = 0.840, *P* < 0.01). Conversely, RVEF showed a significant negative correlation with pulmonary artery systolic pressure (PASP) (r = −0.707, *P* < 0.01). Additionally, TAPSE was significantly positively correlated with both SLS (r = 0.785, *P* < 0.01) and FWLS (r = 0.769, *P* < 0.01). Furthermore, a significant positive correlation was observed between SLS and FWLS (r = 0.806, *P* < 0.01).

### 3.5 Diagnostic efficacy of 3D Auto RV ultrasound parameters for CMS

**Figure 5:**
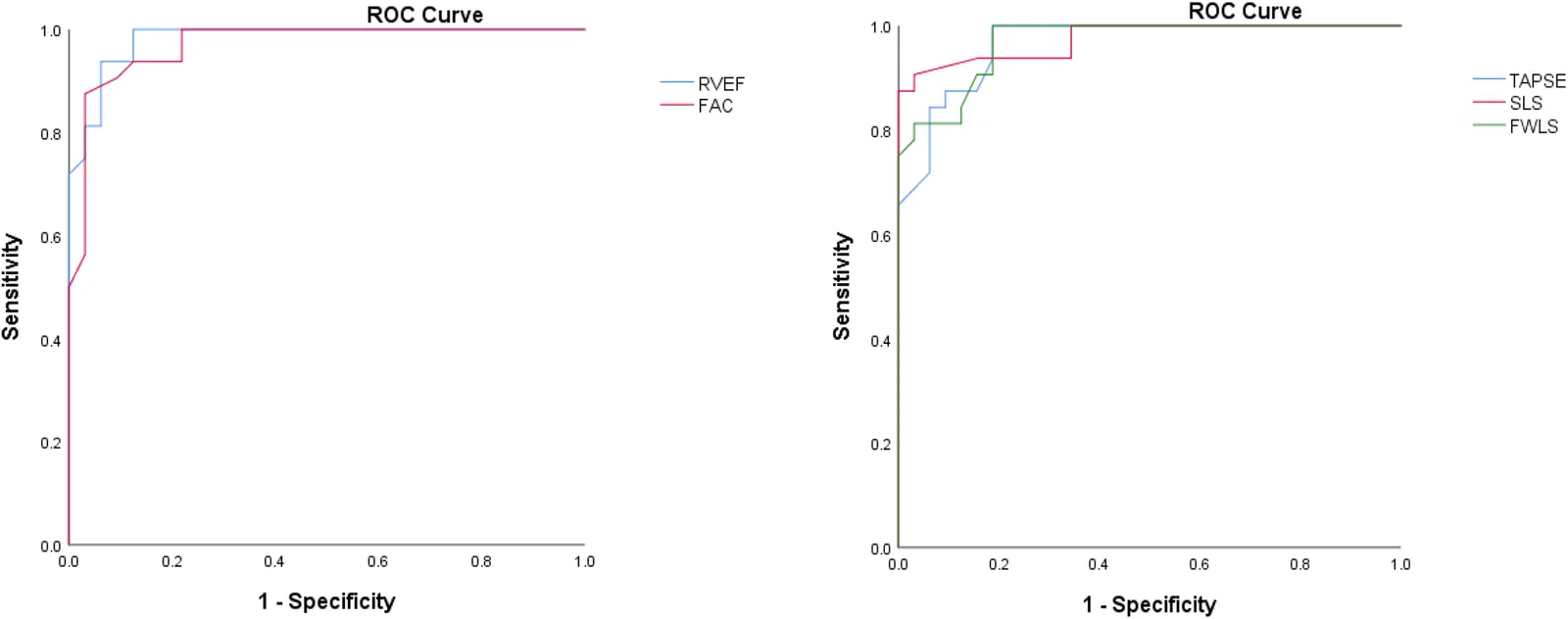
Receiver operating characteristic (ROC) curves of right ventricular systolic function parameters for diagnosing chronic mountain sickness (CMS). Left panel: ROC curves for RVEF (AUC = 0.982) and RVFAC (AUC = 0.970). Right panel: ROC curves for TAPSE (AUC = 0.965), SLS (AUC = 0.975), and FWLS (AUC = 0.968). All parameters demonstrated excellent diagnostic performance, with area under the curve (AUC) values exceeding 0.95.

Table 6 evaluates the diagnostic efficacy of RVEF, RVFAC, TAPSE, SLS, and FWLS for CMS. RVEF shows optimal diagnostic performance at a cutoff value of 46.70. Similarly, RVFAC achieves its best diagnostic efficacy at a cutoff of 36.75, while TAPSE is most effective at 16.20. SLS demonstrates peak diagnostic performance at a cutoff of 21.80, and FWLS is most effective at 19.85 (Figure 5).

**Table 6:** Diagnostic efficacy of 3D Auto RV ultrasound parameters for CMS.

Parameter	AUC	95%CI	Standard error	Sensitivity	Specificity	Cut-off value
RVEF (%)	0.982	0.958–1.000	0.012	0.938	0.937	46.70
RVFAC (%)	0.970	0.934–1.000	0.018	0.875	0.969	36.75
TAPSE (mm)	0.965	0.928–1.000	0.019	1.000	0.812	16.20
SLS (%)	0.975	0.942–1.000	0.017	0.906	0.969	21.8
FWLS (%)	0.968	0.934–1.000	0.017	1.000	0.812	19.85

### 3.6 Repeatability test

Table 7 reveals that the intra-class consistency ICC for RVEF is remarkably high at 0.969, with inter-class consistency also achieving 0.941, underscoring the index's reliability. Similarly, RVFAC, TAPSE, FWLS, and SLS demonstrate comparable high consistency. The findings indicate strong reproducibility for both multiple measurements by the same observer (intra-class consistency) and those conducted by different observers (inter-class consistency).

**Table 7:** Assessment of repeatability and reproducibility of 3D auto RV in measuring right ventricular systolic function in CMS patients.

Intraobserver agreement	ICC	*P*	Interobserver agreement	ICC	*P*
RVEF (%)	0.969	<0.001	RVEF (%)	0.941	<0.001
RVFAC (%)	0.906	<0.001	RVFAC (%)	0.873	<0.001
TAPSE (mm)	0.942	<0.001	TAPSE (mm)	0.881	<0.001
FWLS (%)	0.955	<0.001	FWLS (%)	0.942	<0.001
SLS (%)	0.948	<0.001	SLS (%)	0.933	<0.001

## 4 Discussion

Chronic Mountain Sickness (CMS) is a distinct clinical syndrome primarily affecting individuals residing at altitudes above 2,500 meters (26). Prolonged exposure to high-altitude, low-oxygen environments often results in increased hemoglobin concentration, elevated pulmonary artery pressure, abnormal cerebral blood flow regulation, and blood-brain barrier dysfunction. Persistent elevation of pulmonary artery pressure can lead to right-heart dysfunction or even right-heart failure (27, 28). Numerous studies have shown that declining right-heart function correlates with a higher incidence of adverse cardiovascular events in these patients (14, 15, 29). Consequently, promptly and accurately assessing right-heart function parameters in CMS patients is essential, as it significantly influences clinical diagnosis and treatment decisions.

Currently, conventional echocardiography and cardiac magnetic resonance imaging (MRI) are the primary methods for assessing right heart function. However, MRI is not widely adopted in clinical settings due to its high cost and time requirements. While conventional echocardiography is quick and effective, its accuracy and efficiency are heavily reliant on the operator's expertise and the patient's acoustic window conditions, owing to the limitations of traditional two-dimensional (2D) ultrasound and the complex anatomy of the right heart. To enhance clinical practice and patient care, this study utilized real-time three-dimensional (3D) echocardiographic right ventricular quantification software (3D Auto RV) to assess right ventricular systolic function in patients with chronic mountain sickness (CMS). This technology significantly compensates for the limitations of 2D ultrasound in evaluating right heart function (21, 22, 30) and shows a strong correlation with right heart function parameters measured by cardiac MRI. In this study, we employed the 3D Auto RV system to compare various right heart function parameters between CMS patients and healthy controls, analyzing the diagnostic value and consistency of each parameter. This provides crucial imaging evidence for diagnosing CMS and evaluating right heart function.

The study's results show that in the CMS group, parameters like RVEF, RVFAC, TAPSE, SLS, FWLS, and S’ were significantly lower compared to the control group, while PASP was notably higher. Additionally, RBC, HCT, and HGB levels in the CMS group were significantly elevated relative to the control group, aligning closely with the pathophysiological mechanism of CMS. Long-term hypoxic conditions lead to pulmonary vasoconstriction, remodeling, and a sustained increase in pulmonary artery pressure, which subsequently raises right ventricular afterload, causes myocardial remodeling, and impairs function (31). Additionally, there was a significant difference in MPA between the groups, indicating that the functional and hemodynamic changes in the right ventricle can result in structural alterations in the pulmonary artery, consistent with previous studies (32).

The correlation analysis of ultrasound parameters revealed a strong positive correlation between RVEF and parameters such as SLS, FWLS, RVFAC, TAPSE, and S’, suggesting these measures consistently evaluate right ventricular systolic function. Similar findings have been documented in previous studies (33, 34). Notably, PASP showed a negative correlation with all right-heart function parameters, underscoring the pivotal role of pulmonary hypertension in the decline of right ventricular function (35). This highlights the importance of early monitoring of pulmonary artery pressure and right-heart function in plateau populations.

ROC curve analysis demonstrated that RVEF, RVFAC, TAPSE, SLS, and FWLS exhibited exceptionally high diagnostic efficacy in discriminating CMS patients from healthy controls, with all area under the curve (AUC) values exceeding 0.96. The optimal cutoff value of RVEF for diagnosing CMS was 46.7%, which is highly consistent with the reference threshold for right ventricular dysfunction (RVEF < 47%) recommended by the European Society of Cardiology (ESC) guidelines (26). The ESC guidelines adopt RVEF < 47% as one of the reference standards for right ventricular systolic dysfunction, and the cutoff obtained in our study coincides with this value, further validating the applicability of this threshold in the high-altitude CMS population. However, the ESC guidelines also emphasize that right ventricular function should not be assessed based on a single parameter alone, but rather through a comprehensive evaluation integrating multiple parameters such as TAPSE, FAC, and tissue Doppler S’. In line with this, our study revealed that the optimal cutoff values for TAPSE (16.2 mm, close to the guideline-recommended <17 mm) and RVFAC (36.75%, close to the guideline-recommended <35%) were generally consistent with the recommended thresholds.

A systematic review and meta-analysis included a total of 885 patients with pulmonary arterial hypertension (PH). The results showed that the combined risk ratio of RVEF derived from three-dimensional ultrasound imaging (3DE) was 0.91 (95% confidence interval: 0.85–0.97), which has been confirmed to have significant prognostic value for patients with PH (36, 37). The mean 3DE-derived RVEF in that PH cohort was 35.5 ± 9.07%, which is comparable to the RVEF value (37.65%) observed in our CMS group. As one of the important causes of pulmonary arterial hypertension (PH) in plateau regions, previous studies have shown that CMS exhibits a similar degree of right ventricular dysfunction as that observed in general PH patients (21). Confirmed that RVEF measured by the 3D Auto RV software is highly correlated with the cardiac magnetic resonance (CMR) gold standard, with intraclass correlation coefficients (ICC) exceeding 0.94, providing robust support for the accuracy of this technique in our study.

CMS patients are chronically exposed to hypoxic environments, leading to pulmonary arteriolar constriction, remodeling, and sustained elevation of pulmonary artery pressure, with a progressive increase in right ventricular afterload. Conventional two-dimensional echocardiography is hampered in accurately assessing right ventricular function due to the complex (crescent-shaped and irregular) morphology of the right ventricle. In contrast, the 3D Auto RV technique does not rely on geometric assumptions and can fully capture global volumetric changes of the right ventricle, making it particularly suitable for diseases characterized by chronic right ventricular pressure overload, such as CMS. In the present study, both intra- and inter-observer ICC values for all parameters measured by 3D Auto RV exceeded 0.85, confirming the excellent reproducibility of this technique in CMS patients and establishing a technical foundation for its clinical implementation in high-altitude regions.

The repeatability test results demonstrated that the intra-class and inter-class ICC values for all parameters measured by 3D Auto RV exceeded 0.85. This indicates excellent operational consistency and measurement stability, making the technology suitable for clinical adoption. Its automated analysis feature notably reduces reliance on the operator's experience, addressing the subjectivity and structural limitations inherent in traditional two-dimensional echocardiography for right-heart evaluation. Consequently, it offers a reliable tool for the standardized and quantitative assessment of right-heart function.

In conclusion, 3D Auto RV offers a comprehensive, accurate, and reproducible assessment of right ventricular systolic function in patients with CMS. Among the various parameters, RVEF stands out with exceptionally high diagnostic value, making it a key indicator for clinical screening and follow-up. This study provides crucial imaging evidence for early diagnosis, disease assessment, and monitoring treatment responses in CMS. It also highlights the potential of artificial intelligence-assisted ultrasound technology in high-altitude medicine. Future research should focus on increasing sample sizes, conducting multi-center studies, and integrating long-term follow-up data to further validate each parameter's predictive value for adverse cardiovascular events.

## 5 Conclusions

The study's results indicate that, compared to the healthy population, patients with CMS exhibit lower values in several three-dimensional ultrasound parameters measured by 3D Auto RV quantitative software, including RVEF, TAPSE, RVFAC, SLS, FWLS, and S'. Additionally, these patients show increased PASP and a widened MPA. Furthermore, the CMS group has higher RBC, HCT, and HGB levels than the control group. Specifically, these patients show reduced RVEF, TAPSE, RVFAC, SLS, FWLS, and S', along with increased PASP and a widened MPA. Notably, there are strong pairwise correlations among RVEF, SLS, FWLS, RVFAC, TAPSE, and S’. SLS, FWLS, RVFAC, TAPSE, and S' all positively correlate with RVEF, while PASP negatively correlates with RVEF, SLS, FWLS, TAPSE, and S'. Additionally, the optimal cut-off values for CMS patients, as determined by the 3D Auto RV quantitative software, are 46.7% for RVEF, 36.75% for RVFAC, 16.2 mm for TAPSE, 21.8% for SLS, and 19.85% for FWLS. The intra-class and inter-class consistency ICC values for RVEF, RVFAC, TAPSE, FWLS, and SLS exceed 0.8, demonstrating good repeatability and consistency.

## 6 Limitations

The present study has several acknowledged limitations. First, it was a single-center investigation, with all participants recruited from Qinghai Provincial People's Hospital. The relative homogeneity of the study population may limit the external validity of our findings. Second, the sample size was relatively small (32 patients per group). Although *a priori* power analysis confirmed sufficient statistical power to detect between-group differences in the primary outcome measures, the power for conducting subgroup analyses was insufficient. Third, the absence of reference standard comparisons—specifically cardiac magnetic resonance (CMR) and right heart catheterization (RHC)—precluded direct validation of the agreement between the 3D Auto RV-derived parameters and these gold-standard modalities. Fourth, longitudinal follow-up data for CMS patients were not available; consequently, the prognostic utility of these parameters for predicting adverse outcomes remains to be established. Fifth, we did not assess the graded effects of different altitudes or varying CMS severities on right ventricular functional parameters. Future multicenter studies with larger sample sizes, incorporating CMR and RHC as reference standards and including extended follow-up data, are warranted to further validate the clinical applicability of 3D Auto RV in the diagnosis and management of CMS.

## Data Availability

The original contributions presented in the study are included in the article/Supplementary Material, further inquiries can be directed to the corresponding author.
